# Supplementary material for: Comparative Analysis of the Mitochondrial Genome of Eggplant (Solanum melongena L.) to Identify Cytoplasmic Male Sterility Candidate Genes
Source: Int J Mol Sci. 2024 Sep 9;25(17):9743. doi: 10.3390/ijms25179743 (PMC11396095; doi:10.3390/ijms25179743)

Supplementary Figure. S1 length of repeats statistics for 327A and 327B

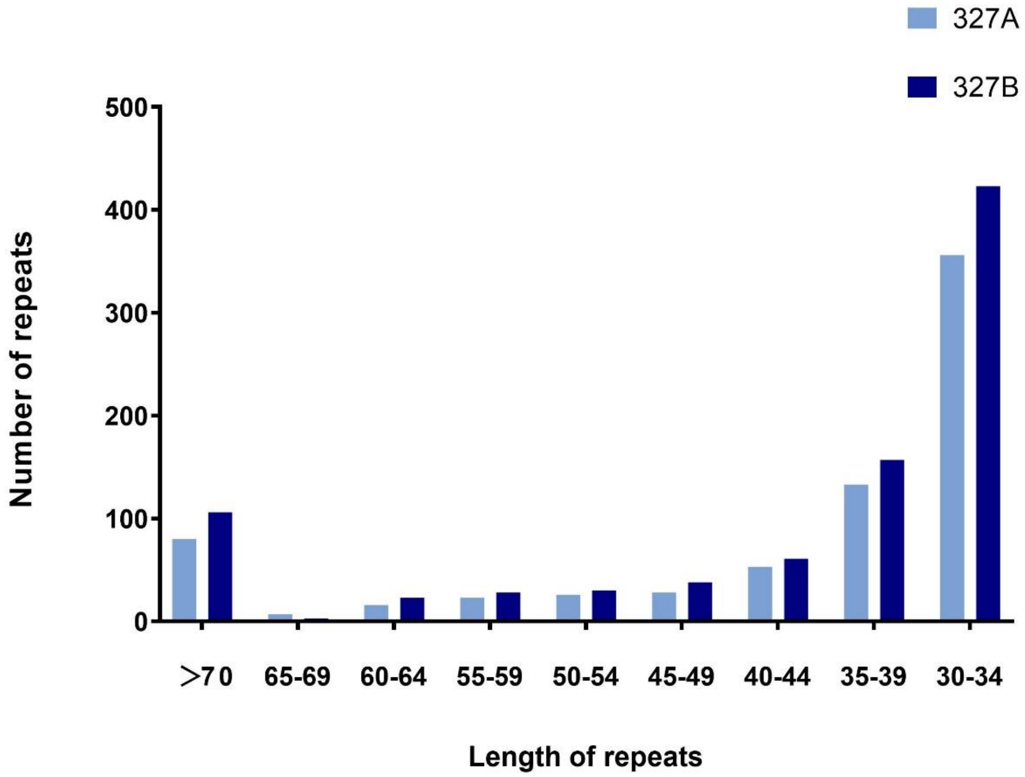

Supplementary Figure. S2 327A and 327B Homologous genes

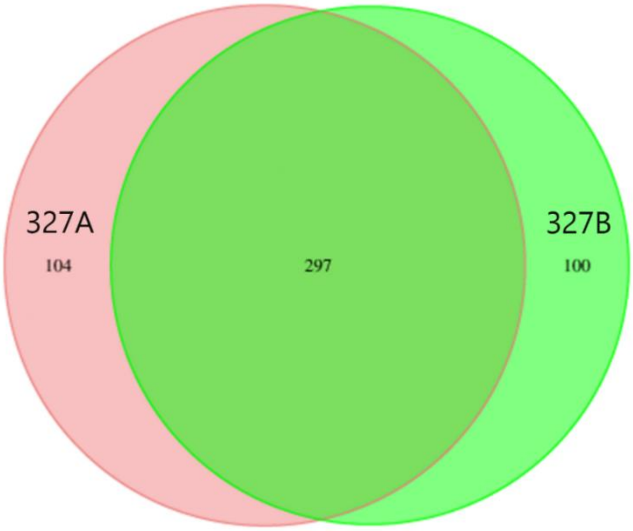

Supplement: Supplementary file 1 [file ijms-25-09743-s001.zip › Supplementary Figure.pdf]
