# Supplementary material for: Comparative Analysis of the Mitochondrial Genome of Eggplant (Solanum melongena L.) to Identify Cytoplasmic Male Sterility Candidate Genes
Source: Int J Mol Sci. 2024 Sep 9;25(17):9743. doi: 10.3390/ijms25179743 (PMC11396095; doi:10.3390/ijms25179743)
Supplement: Supplementary file 1 [file ijms-25-09743-s001.zip › Supplementary table.pdf]

Supplementary Tables

Supplementary Tables S1 Predict the result of 327A and 327B

Product gorup	**327A** Geng ID	Start position （mRNA）	End position (mRNA)	**327B** Geng ID	Start position （mRNA）	End position (mRNA)
Complex I	nad1	11507	184987	nad1	2823	368334
	nad1(chr2)	8988	9372	nad1	2823	185010
	nad2	62683	332890	nad2	76688	342128
	nad2	175274	332890	nad3	306619	306975
	nad3	232329	232685	nad4	156723	165372
	nad4L	279492	279794	nad4	352048	360698
	nad5	5458	77832	nad4L	429052	429354
	nad5	5458	168829	nad5	326958	374386
	nad6	365386	366039	nad5	143035	328591
	nad7	385251	390547	nad6	273849	274502
	nad9	85317	85889	nad7	384904	390200
	nad4L	279492	279794	nad7	127221	132514
				nad9	228513	229085
complex II	sdh3(chr2)	37853	38170	sdh3	17250	17567
	sdh4	280469	280882	sdh4	151432	151845
				sdh4	365576	365989
complex III	cob(chr2)	35194	36375	cob	14591	15772
complex IV	cox1	309390	310973	cox1	54777	56360
	cox2	225244	227410	cox2	106709	108875
	cox3	280810	281607	cox3	150707	151504
				cox3	365917	366714
complex V	atp1	154477	156012	atp1	154481	156016
	atp1	41892	43427	atp1	361405	362940
	atp1	154477	156012	atp4	429543	430139
	atp4	278707	279303	atp6	314034	315332
	atp6	11967	13313	atp8	149599	150069
	atp8	282245	282715	atp8	367352	367822
	atp9	3040	3273	atp9	376571	376804

				atp9	140617	140850
Cytochrome C	ccmB	124256	124876	ccmB	236936	237556
	ccmC	198851	199603	ccmC	198843	199595
	ccmFc	214415	216680	ccmFc	214415	216680
	ccmFN	313616	315385	ccmFN	59003	60772
Ribosome	rps1(chr2)	20798	21532	rps1	481531	482265
	rps3	37562	40240	rps3	423225	425906
	rps4	363682	364704	rps4	275184	276206
	rps10	308085	309222	rps10	53472	54609
	rps12	231903	232280	rps12	307024	307401
	rps13	182333	182683	rps13	182362	182712
	rps19	37264	37548	rps19	391044	391328
	rps19	384132	384416	rps19	422927	423211
	rpl2	114336	117239	rpl2	244591	247498
	rpl5(chr2)	37133	37681	rpl5	16530	17078
	rpl10	117528	118007	rpl10	243823	244302
	rpl16	40131	40673	rpl16	425794	426339
Maturase	matR	69670	71646	matR	333144	335120
Methyltransferase	mttB	377581	378420	mttB	397020	397859
				mttB	416388	417227

Supplementary Table S2 The SNP annotation with 327A as the 327B

Sample ID	CDS					Intergenic	Total
	Start nonsyn	Stop nonsyn	Synonymous	Nonsynonymous	Total_CDS_SNP		
327A	0	0	5	19	23	265	288

Notes： Start_nonsyn: non-synonymous mutations of start codon; Stop_nonsyn: non-synonymous mutation of termination codon;Synonymous:synonymous mutation in the gene region; Nonsynonymous: non-synonymous mutations in the gene region Total_CDS_SNP： mutation in the gene Encoding area ;Intergenic： SNP in the intergenic region

Supplementary Table S3 Annotate gene mutations for 327A

Geng ID	mutate_type	ref_codon <->sample_codon	ref_aa <->sample_aa
cox2	syn	CCT<->CCG	P<->P
cob	syn	ATA<->ATT	I<->I
rpl2	syn	GTT<->GTA	V<->V
nad4L	syn	ATA<->ATC	I<->I
sdh3	nonsyn	TTC<->TGC	F<->C
sdh3	nonsyn	TCC<->TGC	S<->C
sdh3	nonsyn	AAG<->AAT	K<->N
ccmFc	nonsyn	CAA<->AAA	Q<->K
rpl10	nonsyn	ACA<->AAA	T<->K
atp6	nonsyn	CCC<->CTC	P<->L
rpl16	nonsyn	CGT<->AGT	R<->S
rpl16	nonsyn	CGA<->CTA	R<->L

rpl16	nonsyn	AAT<->AAG	N<->K
rpl16	nonsyn	TCA<->GCA	S<->A
rps1	nonsyn	CGG<->CAG	R<->Q

Notes：ref:reference=327B；sample=327A；syn: synonymous mutation；nonsyn: Nonsynonymous mutation ；ref_codon<->sample_codon：The reference sequence in which the SNP resides triplet codon <-> sample triplet codon；ref_aa<->sample_aa：The reference sequence of the SNP is amino acids <-> sample amino acids.

Supplementary Table S4 The indel annotation with 327A as the 327B

Sample ID	Insertion	Deletion	CDS_InDel	Total
327A	28	58	0	86

Notes：CDS_InDel : mutation in the gene Encoding area

Supplementary Table S4 characteristic ORF（open reading frame）in 327A and 327B

327A characteristic ORF			327B characteristic ORF		
orf100b	orf181b	orf77k	orf101d	orf155a	orf75a
orf100c	orf193a	orf78n	orf103d	orf161b	orf75g
orf101f	orf199a	orf78o	orf104d	orf165a	orf75k
orf101g	orf204a	orf79l	orf105a	orf165b	orf78g
orf102d	orf208b	orf79m	orf105d	orf185a-1	orf78m
orf104e	orf215a	orf79n	orf105g	orf185a-2	orf80a
orf106d	orf217a	orf79o	orf106b	orf195a	orf80c
orf106e	orf219a	orf79p	orf106c	orf209a	orf80d
orf106f	orf228a	orf80k	orf107a	orf216a	orf80g
orf107d	orf244a	orf80l	orf107c	orf218a-1	orf80h
orf107e	orf260a	orf80m	orf109c	orf218a-2	orf80i

orf108d	orf265a	orf81j	orf111a	orf227a-1	orf81d
orf110d	orf269a-1	orf82j	orf113a	orf227a-2	orf83b
orf113b-1	orf269a-2	orf82k	orf114a	orf274a-1	orf85i
orf113b-2	orf312a	orf82l	orf115b	orf274a-2	orf85j
orf114c	orf314a	orf82m	orf117a	orf294b	orf85k
orf118c	orf327a	orf83i	orf121a	orf295a	orf86e
orf118d	orf379a	orf84f	orf121b	orf303a	orf87f
orf118e	orf443a	orf84g	orf123b	orf381a	orf88a
orf120b	orf509b	orf85m	orf125a	orf594a	orf88d
orf123c	orf71m	orf86h	orf126a	orf71d-1	orf88e
orf129c	orf71n	orf88g	orf126b	orf71d-2	orf89b
orf130c	orf71o	orf895a	orf126c	orf71g	orf91a
orf131c	orf71p	orf89d	orf131a	orf72a	orf91c
orf137b	orf72n	orf90i	orf135b	orf72j	orf91e
orf152b	orf72o	orf90j	orf136a-1	orf72k	orf92c
orf168a	orf72p	orf91g	orf136a-2	orf72m	orf92i
orf169b	orf73k	orf92j	orf141a	orf73g	orf933a-1
orf169c	orf73l	orf93l	orf148a-1	orf74a	orf933a-2
orf172a	orf73m	orf94e	orf148a-2	orf74b	orf93b
orf178a	orf73n	orf95d	orf151a	orf74f	orf93f
orf180a	orf75m	orf95e	orf152a-1	orf74g	orf93g-1
orf181a	orf76k	orf96e	orf152a-2	orf74o	orf93g-2
orf181b	orf76l	orf96f	orf154a-1	orf74p	orf96b
orf99h			orf154a-2	orf74r	orf96c
			orf96c	orf97b	orf97f
			orf96d	orf97d	orf98a-1
			orf97a	orf97e	orf98a-2
			orf99b	orf99f	orf99g

Supplementary Table S5 The open reading frames(ORFS) more than 100 amino acids in 327A unique areas

Geng ID	strand	mRNA start(bp)	mRNA end(bp)	No. of transmembrane domain
orf603a	+	313574	315385	3
orf102c	+	319545	319853	1
orf1579a	+	321827	326566	0
orf130c	+	342544	342936	0
orf188a	+	343263	343829	0
orf110c	+	347112	347444	1
orf112a	-	257636	257974	1
orf322a	-	227722	228690	1
orf327a	-	229009	229992	0
orf110b	-	231281	231613	1
orf269a-2	-	232811	233620	3
orf106b	-	284858	285178	1
orf252a	+	12555	13313	5
orf116a	+	14450	14800	0
orf205a	+	21507	22124	0
orf379a	+	22021	23160	0
orf113a-2	-	43488	43829	0
orf115a	-	51052	51399	0
orf103a-2	-	54154	54465	0
orf132a-2	-	58987	59385	0
orf138a	-	72883	73299	0
orf107b	-	77743	78066	0
orf181a	-	78023	78568	2
orf165a	-	78819	79316	0
orf109b	-	8460	8789	0
orf118b	-	387511	387867	0
orf961a	-	134636	137521	0
orf443a	+	370703	372034	7
orf561a	+	38555	40240	0
orf312a	+	40613	41551	3
orf234a	-	248109	248813	1

orf322a	-	227722	228690	1
orf109b	-	8460	8789	0
orf152a	+	23450	2390	0
orf314a	+	22316	23260	3
orf172a	-	46826	47344	3
orf119a	+	262314	262673	0
orf101c	+	264382	264687	0
orf118a	+	264820	265176	0

Supplementary Table S6 Oligonucleotides used in this study

Primer names	Forward (5' – 3')	Reverse (5' – 3')	Application
Orf561a-rpl16-orf312a	TATCAAGCAAGAAGGAAGCGGC	GCGGAGTTCTCTCAGGTCTT	RT-PCR
rpl16	ATGCTCCTGCGGAAGTAT	GGTACGACCACCGAACGAA	RT-PCR
Orf172a	TCAAGGTATTCCGGGAGA	ATGACTATCATTCGTTATTTTTTT	RT-PCR
Orf443a	ATGTATAGGACCAACTGGGG	CCTACTGCAATAATTCTTGCT	RT-PCR
Orf312a	AAGTTCGTTCGGTGGTCGTA	CGTGGTCATTATAGCGGTTC	real-time PCR
rpl16	TGAGCGGACAATTCCGAAGA	ACGAGCAATCCAACCCGTAG	real-time PCR
Orf172a	GCCCTTCCAACCCATCAAAC	TTCACAGGGAGGTGATTTCG	real-time PCR
Orf443a	AGGCATCCCAGGTAATTCAG	AATAACCACGTCCGCTGAAT	real-time PCR
